# Strengthening responses to dementia: Building an evidence platform for the development of Vietnam’s National Dementia Plan

**DOI:** 10.1002/alz.086502

**Published:** 2025-01-09

**Authors:** Tuan Anh Nguyen, Thu Ha Dang

**Affiliations:** ^1^ National Ageing Research Institute, Melbourne, VIC Australia

## Abstract

**Background:**

Prior to formulating an effective national dementia plan in Vietnam, reliable information on current situation of dementia care, treatment and support in Vietnam was needed. This study was conducted to describe Vietnam’s response to dementia at three levels: individual (consumers and providers); health and social care organisations; and national healthcare system to guide the plan development.

**Method:**

Guided by the “Towards a dementia plan: a WHO guide”, a comprehensive situational analysis was conducted, focusing on Policy, Service Delivery and Epidemiological assessments.

**Result:**

Vietnam had a high level institutional and policy framework on aging, NCD, mental health and disability including the 2009 Law on the Elderly, which provides the legal umbrella for policies on older people. However, no dementia‐specific policy existed and policies to promote healthy brain remained weak. Rapid aging significantly contributes to the explosion of NCD including dementia in Vietnam. There were 660000 Vietnamese people estimated to be living with dementia in 2015, with resultant dementia related costs of US$ 960 million. The healthcare system is not yet prepared for the shift to NCD from an acute, communicable disease burden in the past. Health service delivery is hospital‐centric, with over‐reliance on hospitals and under‐utilization of primary care system that in turn is fragmented and poorly prepared to address the rising challenge of dementia. Both the public and primary healthcare providers have poor knowledge and little confidence but more positive attitudes toward dementia care and management. Social care and support specific for dementia is lacking although there is an impressive grassroots organisation of older people with nearly 100,000 branches.

**Conclusion:**

To allow for a more harmonized response across the health sector and more effective use of limited resources, an integrated national action plan for dementia is sensible. However, Vietnam should take into consideration the potential for fragmentation and lack of dedicated resources being allocated to dementia. A new, integrated model of care focusing on a stronger primary healthcare system, community‐based social care and a healthy aging approach is needed to improve dementia prevention, care, treatment and support in Vietnam.

